# The Molecular Mechanisms of Complement Receptor 1—It Is Complicated

**DOI:** 10.3390/biom13101522

**Published:** 2023-10-13

**Authors:** Matthew P. Hardy, Mariam Mansour, Tony Rowe, Sandra Wymann

**Affiliations:** 1 CSL, Bio21 Institute, Melbourne, VIC 3052, Australia; 2 CSL, CSL Biologics Research Centre, 1066 Bern, Switzerland

**Keywords:** soluble, complement, receptor, CSL040, mechanism, domain

## Abstract

Human complement receptor 1 (CR1) is a membrane-bound regulator of complement that has been the subject of recent attempts to generate soluble therapeutic compounds comprising different fragments of its extracellular domain. This review will focus on the extracellular domain of CR1 and detail how its highly duplicated domains work both separately and together to mediate binding to its main ligands C3b and C4b, and to inhibit the classical, lectin, and alternative pathways of the complement cascade via the mechanisms of decay acceleration activity (DAA) and co-factor activity (CFA). Understanding the molecular basis of CR1 activity is made more complicated by the presence not only of multiple ligand binding domains within CR1 but also the fact that C3b and C4b can interact with CR1 as both monomers, dimers, and heterodimers. Evidence for the interaction of CR1 with additional ligands such as C1q will also be reviewed. Finally, we will bring the mechanistic understanding of CR1 activity together to provide an explanation for the differential complement pathway inhibition recently observed with CSL040, a soluble CR1-based therapeutic candidate in pre-clinical development.

## 1. Introduction

**Figure 1:**
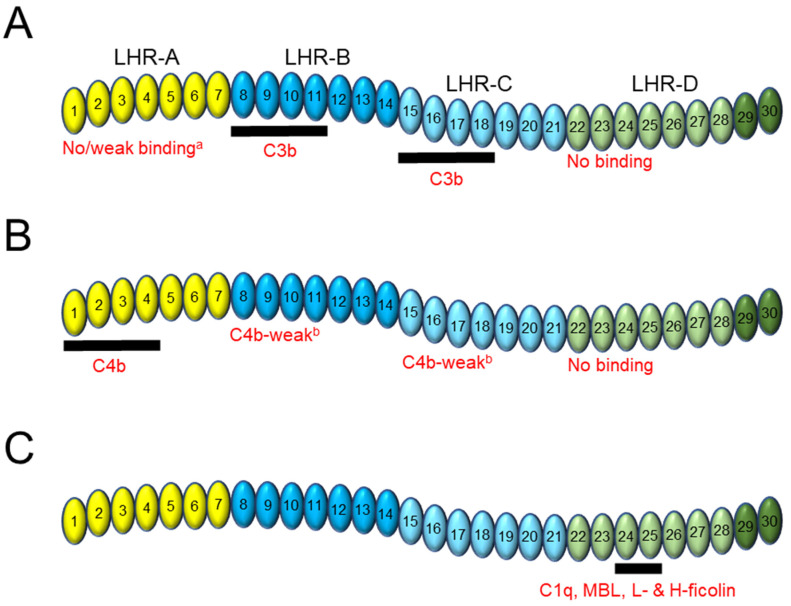
The contribution of CR1 domains to ligand binding. Shown schematically is the extracellular domain of CR1 made up of four long homologous repeat (LHR) domains, A to D. Each LHR domain (identified by a particular color) is itself comprised of 7 short consensus repeat (SCR) domains (numbered). LHR-B and LHR-C are highly homologous and so are shaded in similar colors to show their functional similarity. SCR29–30 (shaded in a darker green than SCR22–28) are not technically part of LHR-D but are included for the purposes of this review. The extracellular domain of CR1 is also known as soluble CR1 (sCR1). Beneath each schema is shown the LHR and SCR contributions to (**A**) C3b binding, (**B**) C4b binding, and (**C**) C1q, mannose-binding lectin (MBL), L-ficolin, and H-ficolin binding. The thick horizontal black bar lies beneath the SCR domains responsible for the binding interaction. ^a^ Several studies show no binding of LHR-A to C3b; other experiments show weak binding (<10% relative to LHR-ABCD).^b^ Binding observed in the absence of LHR-A.

Before we take a close look at the molecular basis of CR1 function, it is worth briefly describing the molecule itself and the milieu in which it resides. CR1 is a type I membrane glycoprotein expressed on the surface of erythrocytes (E-CR1) and immune cells that acts as a central regulator of the classical, lectin, and alternative pathways of the complement system [1,2,3,4]. The complement system is an integral part of the innate immune response and has been extensively reviewed [5,6,7,8,9,10]. The predominant allelic variant of CR1 (CR1*1) that we will be discussing here has a large, flexible extracellular domain comprised of 30 highly homologous domains called short consensus repeats (SCRs), followed by a transmembrane domain and a short, 43-amino acid cytoplasmic tail [11,12]. SCR domains 1–28 are arranged in groups of seven to form four larger units: long homologous repeat (LHR) domains -A to -D (Figure 1A) [11,13,14,15]. Including SCR domains 29–30 into an expanded definition of LHR-D has been performed routinely [16,17,18] and will be retained for the purposes of this review. Soluble versions of CR1 containing varying extracellular domain fragments have been generated for intended therapeutic use, of which CSL040 is a recent example [19]. In this article, we will review the contribution of CR1 and its extracellular LHR and SCR domains to ligand binding, DAA, CFA, and inhibition of the three complement pathways.

## 2. CR1 Binding to C3b and C4b

### 2.1. General and Comparative

CR1 was first described as the receptor for C3b following a series of experiments that identified a 205,000 Dalton molecular weight glycoprotein from solubilized membrane fractions extracted from erythrocytes and immune cells with the ability to promote cleavage of C3b by complement factor I and to displace complement factor Bb from the alternative pathway C3 convertase [1,20]. Subsequent in vitro experiments using a variety of techniques such as radio-ligand binding assays and surface plasmon resonance (SPR) performed on both cell surface and soluble CR1 (sCR1) were able to estimate the affinity of the interaction to C3b in greater detail. This included both monomeric and dimeric forms of C3b, and the results are summarized in Table 1. Soluble CR1 (sCR1) refers to the entire extracellular domain of CR1 (Figure 1) and usually describes a form derived recombinantly, since levels of endogenous sCR1 produced by proteolytic shedding from the cell surface are very low [21].

Despite some variation in the experimental results due to the nature of the techniques employed, it is evident that CR1 binds to dimeric C3b with low nanomolar affinity (Table 1). This affinity is significantly stronger than the affinity of CR1 to monomeric C3b, suggesting a bivalent interaction of CR1 with dimeric C3b, leading to an increased avidity that has been noted by others [24,26]. Affinities generated to plasma-derived C3b [18,30] are generally weaker than earlier measurements using C3b fractions, possibly reflecting a preference for the interaction of CR1 with the predominantly monomeric C3b in the preparation, as the affinities are similar (Table 1). Several early studies have shown no binding of CR1 to iC3b [2,31], but more recent work using SPR techniques showed weak but detectable binding [29], which makes sense when the ability of complement factor I to cleave iC3b in the presence of CR1 to C3c and C3dg is considered [24,32]. No binding of CR1 to C3d under physiological conditions has been measured [30].

The first published reference to the binding specificity of CR1 to C4b was in 1980 [33]. Later studies confirmed this interaction [34,35], with one study also showing no difference in CR1 binding to C4b alone compared to C4bC2a (C4bC2b using current nomenclature) whereas a two-fold increase in affinity to C3bBb compared to C3b (despite very weak binding to factor Bb alone) was observed [35]. There is surprisingly little affinity data on the interaction between CR1 and C4b. However, from the available binding data (summarized in Table 2), some conclusions can be made. Firstly, the affinity of CR1 to C4b—monomer or dimer—is conservatively at least 10-fold weaker than the affinity between CR1 and C3b. This has implications for binding site usage within the LHR domains of CR1 (as discussed later), where there may be a preference for binding to C3b where binding sites for both C3b and C4b exist [36]. Secondly, and similar to the phenomenon observed for C3b, the affinity of CR1 to dimeric C4b is also substantially stronger than it is to monomeric C4b, suggesting that like C3b, the interaction between CR1 and C4b dimer is also bivalent, resulting in increased avidity [36] (Table 2).

In contrast to C3, C4 zymogen exists as two structurally similar isoforms, C4A and C4B [37]. While some early experiments showed increased binding of CR1 to activated C4Ab compared to C4Bb, a more recent experiment using SPR showed no quantitative difference in affinity between CR1 and either isoform of C4b [38,39,40]. With regards to the cleavage products of C4b, only a weak (~1.6 μM) affinity of CR1 to C4c has been demonstrated, with no binding observed to C4dg [38]. Finally, comparative competition experiments showed that approximately 10-fold more sCR1 was required to achieve 50% inhibition of the interaction of ^125^I-labelled C4b dimer (C4-ma, a C4b analog, was used) with E-CR1 compared to C3b dimer [24]. This further highlights the strength of the binding of CR1 to C3b relative to C4b.

**Table 2:** Binding of CR1 to C4b.

Affinity to C4b Dimer (nM)	Affinity to C4b Monomer (nM)	Method Used	CR1 Source	References
12 ^a^	>100-fold weaker ^b^	Radioligand binding/competition	sCR1 and E-CR1	Wiesman et al., 1990 [24]
340	>100-fold weaker ^b^	Radioligand binding assay	E-CR1	Reilly et al., 1994 [36]
360	>100-fold weaker ^b^	Radioligand binding assay	Expressed on CHO cells	Reilly et al., 1994 [36]
120–480 ^c^	Not measured	Radioligand binding assay	E-CR1	Reilly and Mold 1997 [40]
330–390 ^d^	900	SPR	sCR1	Clemenza and Isenman 2004 [38]

^a^ Affinity estimate based on the concentration of sCR1 needed for 50% inhibition of C4-ma (C4b-like) binding to E-CR1. ^b^ Compared to dimer. ^c^ Affinity of CR1 to C4b dimer (A isoform) measured at 120–140 nM; affinity to C4b dimer (B isoform) measured at 410–480 nM. ^d^ In total, 13% of the binding sites were determined to be of high affinity (8–12 nM). E-CR1: erythrocyte-expressed CR1. CHO: Chinese hamster ovary. SPR: surface plasmon resonance.

In conclusion, CR1 binds both dimeric C3b/C4b with substantially higher affinity than it does to monomeric C3b/C4b, suggesting differences in the nature of the interaction—monovalent versus bivalent. Secondly, the CR1–C3b interaction is significantly stronger than the CR1–C4b interaction, which has implications for ligand binding preferences and the relative strength of complement pathway inhibition by CR1 and its sCR1 variants.

### 2.2. Domain Contribution

Initial attempts to unravel the domain contribution of CR1 to C3b binding were relatively crude, utilizing proteolytic digestion of CR1 and immunoblotting to show the involvement of the N-terminal half of CR1 in C3b binding [41]. This was followed by experiments using single CR1 LHR domains or combinations thereof to build a more complete picture of each domain’s interaction with C3b, as shown in Figure 1A. Despite some experiments suggesting weak, qualitative binding of the LHR-A domain of CR1 to C3b (1–10% compared to LHR-ABCD) [34,42,43], other data showed no interaction [27,44,45]. For example, Scesney et al. [27] generated a version of sCR1 lacking the LHR-A domain (desLHR-A/LHR-BCD) and showed that it competed equally with LHR-ABCD for the binding of dimeric C3b to E-CR1. Additional studies might help to clarify the question of C3b binding affinity (as a monomer and/or dimer) to the LHR-A domain of CR1 since this domain appears to contribute significantly to the ability of sCR1 to inhibit alternative pathway activity as discussed below [18]. No evidence for the binding of LHR-D—with or without SCR29–30—to C3b has been reported [26,34,45].

There is strong evidence that LHR-B [16,27,34,41,42,43,45] and LHR-C [16,25,27,34,45,46] are the primary domains responsible for C3b binding. Klickstein et al. [34] were the first to demonstrate LHR-B and LHR-C binding to C3b, using LHR-BD and -CD truncation mutants to show similar C3b rosette formation (an in vitro assay where cellular clusters—“rosettes”—are formed by CR1-expressing cells bound to and surrounded by C3b-coated erythrocytes and visualized microscopically) compared to LHR-ABCD. However, subsequent experiments suggested that both LHR-B and -C are required to achieve full C3b binding, specifically the dimeric form. In one experiment, both LHR-BD and -CD mutants stably expressed on K562 cells and assayed for binding to C3b dimer showed dissociation constants of between 2.0 and 2.5 nM compared to only 1 nM for LHR-ABCD [16]. Another experiment showed a 10-fold decrease in the molar concentration of a recombinant LHR-ACD variant (lacking LHR-B) to inhibit the uptake of ^125^I-C3b dimer by E-CR1 compared to recombinant LHR-ABCD/sCR1 [25]. In contrast, no differences were observed for monomeric C3b.

Further refinement of the C3b epitope on CR1 to individual SCR domains within both LHR-B and -C has been performed, and it has been suggested that SCR8–10 and SCR15–17 of LHR-B and -C, respectively, comprise the binding site for C3b [11,15]. However, this may need to be redefined to encompass SCR8–11 and SCR15–18 based upon a reappraisal of key data. For example, immunofluorescence experiments using transiently expressed COS cells demonstrated [16] that SCR8–11 and SCR15–18 of LHR-B and -C, respectively, showed comparable (96%) C3b binding to the entire LHR domain. The first three SCR domains showed only 66% C3b binding, and this was further reduced to no detectable binding with only two SCR domains. This was followed by radioligand binding assays, which also showed comparable C3b affinity (1.8–2.4 nM) of SCR8–11 to LHR-B, and SCR15–18 for LHR-C [16]; this affinity for C3b was weaker at 6.2–8.7 nM against SCR8–10 or SCR15–17. A later study also showed SCR8–10 of LHR-B with a 10–30% reduction in ligand binding compared to LHR-B alone [43].

In contrast to C3b, the interaction between CR1 and C4b takes place primarily in the LHR-A domain [34,36,42,43,44,45] with some contribution of the LHR-B and -C domains [34,36,42,43,45,46], and no contribution of the LHR-D domain [34,36,45] (Figure 1B). The different specificity of LHR-A ligand can also be explained by amino acid differences between the domains—SCR1–3 shares only 61% identity with SCR8–10 and SCR15–17 [11]. In the same set of experiments described above by Klickstein et al. [34], an LHR-AD chimera showed a similar degree of C4b rosette formation as LHR-ABCD, with LHR-BD and LHR-CD constructs showing a 50–80% reduction in activity and no activity for LHR-D alone. Two subsequent studies [42,44] used immunoprecipitation and ELISA-based experiments to show the interaction of a construct called CR1–4 encoding LHR-A (plus SCR8 and a portion of SCR9) with C4b. Point mutations introduced into this construct within LHR-A (at G76 and Y135) eliminated C4b binding, and another double mutation at R105/N106 significantly reduced binding, confirming LHR-A as the primary C4b binding site. In another study, LHR-ABCD, LHR-ACD, and SCR1–4 fused to LHR-D were shown to have a similar affinity to C4b dimer, suggesting that LHR-A (and specifically SCR1–4), but not LHR-B and -C, binds C4b [36].

In the absence of LHR-A, a weak affinity of 1.2 μM for C4b was measured using a construct encoding SCR15–18 of LHR-C. Other experiments also showed an interaction of LHR-B and/or LHR-C in the absence of LHR-A with C4b, but the experimental results were qualitative [45,46]. Smith et al. [46], however, were able to confirm the specificity of this interaction by showing that a triple point mutation (K912E/K914E/R933E) within LHR-C could abrogate binding to C4b-sepharose compared to unmodified LHR-C. As previously stated, these data suggest that LHR-B and -C binding to C4b is unlikely when competition for binding with C3b is required. In addition, and as observed with C3b, the first four SCR domains within LHR-A may be considered as the full binding motif for C4b, with SCR1–3 showing a 10–30% decrease in binding to C4b compared to LHR-A alone [43].

To summarize the CR1 domain contribution to ligand binding: LHR-A predominantly binds C4b, and LHR-B and LHR-C bind C3b with LHR-D showing no binding to either ligand. For both ligands, full binding requires the first four SCR sub-domains of the respective LHR domain, rather than three. In the absence of LHR-A, C4b can bind LHR-B or -C but the interaction is of weak affinity. Both LHR-B and -C are also required for full (bivalent) C3b dimer binding as the removal of either domain reduces the affinity of interaction. Given that the C3 convertases consist of monomeric ligand and the C5 convertases consist of homo- or hetero-dimeric ligand, it is reasonable to hypothesize that in the former case, the interaction with CR1 is monovalent and in the latter case the flexible nature of CR1 and the spacing between ligand binding sites allows for multiple LHR domains to be engaged to create a high avidity interaction [12,24].

## 3. CR1 Binding to Other Ligands

Far from being a useless appendage at the C-terminal end of the extracellular domain of CR1, the LHR-D domain is also involved in the binding of its own distinct ligands (Figure 1C). Like C3b and C4b, the complement classical pathway component C1q is an opsonin that binds to apoptotic cells and mediates their clearance [47,48]. C1q has also been shown to specifically bind CR1 with high affinity [28], and subsequent experiments using CR1 deletion mutants localized the interaction to LHR-D. Another study soon afterwards showed that the association of C1q with CR1 could occur independently of C3b and C4b binding [49]. More recently, the epitope for C1q on the LHR-D domain of CR1 was mapped to SCR24–25, using a series of truncation and deletion variants [50].

Mannose-binding lectin (MBL), referred to as both an opsonin and a pattern recognition molecule, closely related to C1q and an initiating factor of the complement lectin pathway, was the second ligand discovered to specifically bind the CR1 LHR-D domain [51]. MBL and C1q have similar affinities to CR1 and compete for binding, suggesting an overlapping epitope [51]. A 2013 study [52] confirmed the MBL binding site on CR1 to be similar to that for C1q at SCR24–25 within LHR-D. The same authors identified additional ligands sharing the same binding site on SCR24–45 of CR1—the opsonin and pattern recognition molecules L-ficolin and (weakly) H-ficolin. L-ficolin itself binds to a wide variety of targets and can also induce the activation of the lectin complement pathway [53]. It thus appears that CR1 is a receptor for the main complement opsonins via binding to its LHR-D domain and these interactions may facilitate uptake and removal of immune complexes [54,55].

## 4. Structural Data

Given the long, flexible, nature of the extracellular domain of CR1, which is analogous to a string of pearls, high-resolution structural information on the entire molecule and its interaction with ligand has not been able to be deduced. However, it has been possible to use those specific fragments of CR1 involved in ligand binding for structural studies, most notably for C3b but not C4b. Smith et al. [46] were the first to determine a medium-resolution structure of the C3b/C4b binding domains SCR15–17 (in LHR-C) by nuclear magnetic resonance techniques. The authors were then able to map onto the structure the various residues determined from previous mutagenesis to be involved in C3b and/or C4b binding [46]. Conversely, Janssen et al. [56] determined the crystal structure of C3b to 4 Å and then mapped putative CR1 binding sites onto it across three distinct sites. A medium–low-resolution structure (24 Å) of CR1 SCR1–3 has also been derived, but no C3b/C4b binding information has been described, as the study was concerned with its binding to a malarial protein [57].

A higher resolution crystal structure of C3b bound to CR1 domains SCR15–17 has more recently been described [58], providing detailed information on residues important for this interaction. Unfortunately, these studies only looked at receptor–ligand binding through the lens of a single interaction using a minimal binding fragment of CR1 and were not able to provide a holistic view of how CR1 interacts with multiple ligands (both monovalently and bivalently) across multiple sites. There has been one attempt to determine the structure of the entirety of soluble CR1 [12]. This was a medium-resolution model generated following small-angle X-ray scattering and analytical ultracentrifugation that although did not show the structure of CR1 at the amino acid level, did demonstrate that CR1 was flexible, extended, and could fold back on itself. This, along with a comparison of the relative sizes of C3b and CR1, suggested that CR1 has the potential to simultaneously bind up to three C3b-sized monomeric ligands across LHR-A, -B and -C (plus C1q/MBL in LHR-D) or combinations of monomer and dimer. More work needs to be undertaken to understand these complex interactions in more detail.

## 5. Decay Acceleration Activity of CR1

### 5.1. Classical/Lectin Pathway C3 and C5 Convertases

CR1 inhibits the formation of both the classical (and by inference the lectin) C3 convertase, consisting of a C4bC2b complex, and the classical C5 convertase, which consists of a C4bC2bC3b complex. The mechanism by which this decay acceleration activity (DAA) occurs is through the interaction of CR1 with C4b bound to cell surfaces, which displaces C2b from their shared binding site on C4b [59]. The classical C5 convertase can contain C3b bound covalently as a heterodimer to C4b [60].

The use of deletion mutants of CR1 has been critical to our understanding of how CR1 exerts DAA on the classical C3 and C5 convertases. For inhibition of the classical C3 convertase, both the LHR-A domain and one of either the LHR-B or -C domains are required, although the LHR-A domain appears to be the most important (Table 3). Two studies [25,61] showed that LHR-AC and LHR-ACD deletion mutants had similar classical C3 convertase DAA as compared to LHR-ABCD (Table 3), demonstrating that one of two C3b binding sites on CR1 as well as LHR-D are dispensable. Constructs encoding LHR-B, LHR-C, or combined LHR-BC domains exhibited residual DAA (8–12%) compared to LHR-A or LHR-ABCD [61,62] (Table 3), which correlates with the weak C4b binding described above for these domains and demonstrates the importance of the LHR-A domain. The CR1 LHR-A domain alone was found to have between 40 and 60% DAA compared experimentally to LHR-ABCD (Table 3), which comprises the bulk of activity but is clearly insufficient for full DAA [61,62]. These data therefore suggest situations for classical C3 convertase DAA where the LHR-A domain of CR1 acts on convertases containing monomeric C4b, and LHR-A with either LHR-B or -C domains binding bivalently to dimeric C4b for an additive effect. This makes sense given both monomeric and dimeric forms of C4b are present in plasma and in the classical C3 convertase [18,62].

When we consider the roles of the CR1 LHR domains in driving DAA of the classical C5 convertase, there are some key differences compared to the classical C3 convertase. It was demonstrated that a C3b binding site (LHR-B or -C) could be removed without affecting classical C5 convertase DAA [25,61] (Table 4). However, constructs encoding LHR-A or LHR-BC showed almost no activity compared to LHR-ABCD or LHR-AC [61,62], suggesting that LHR-A with LHR-B or -C act synergistically to provide full DAA of the classical C5 convertase (Table 4). This is likely to involve bivalent binding of LHR-A to C4b and LHR-B or -C to C3b within the C4bC2bC3b convertase complex [61]. Spacing between LHR domains and thus between C4b/C3b binding sites is also important, with experiments using only SCR1–3 of LHR-A fused directly to LHR-C showing a 100-fold drop in classical C5 convertase DAA compared to LHR-AC [62]. Clearly, the number of SCR domains within each LHR domain of CR1 allows the required flexibility for correct folding back of the molecule and bivalent binding to ligand without steric hindrance [12].

### 5.2. Alternative Pathway C3 and C5 Convertases

As with the classical C3 and C5 convertases, CR1 has been shown to mediate decay of the alternative pathway C3 convertase (C3bBb) and C5 convertase (C3bBbC3b) by competitive displacement of Factor Bb from the complexes [1,25,63]. Interestingly, and in line with its relative affinities to C3b and C4b, up to 10-fold more sCR1 is required for DAA of the classical convertases compared to the alternative convertases [25], suggesting a more potent effect on DAA of the alternative pathway.

The contribution of CR1 domains to the DAA of the alternative pathway C3 convertase is not well understood and confounded by spontaneous C3b dimer formation contaminating the experimental results previously used to examine C3 convertase DAA. Instead of C3b monomers, the presence of C3b dimers in the assay means that the results could be reflective of the output of an alternative C5 convertase DAA [61]. When an attempt to address this issue was performed, the bulk (95%) of the DAA of the C3 convertase appeared to be mediated by the LHR-A domain, with only minor (12–14%) contributions measured for the LHR-B and LHR-C domains [61]. This finding is hard to reconcile with the C3b ligand binding data for these domains described above, which show the reverse contributions of these same domains. CR1 binding to C3b monomer as would be found on a C3bBb convertase is much weaker than to dimer (Table 1), and so an affinity of C3b monomer to LHR-A would need to be convincingly demonstrated for the DAA to C3bBb to be properly understood. More work needs to be undertaken before a clear mechanistic explanation can be provided for the DAA of CR1 on the alternative C3 convertase.

The domain contribution of CR1 to the DAA of the alternative pathway C5 convertase is better understood, although again the role of the LHR-A domain is unclear given the ambiguity of the available C3b binding data. What is clear is that LHR-A, -B, and -C domains are all required and act synergistically to provide full activity of sCR1 (Table 5). If either LHR-B or LHR-C is removed, there is a significant drop in alternative pathway C5 DAA. The extent of this drop is uncertain, with one study showing a 30-fold decrease in DAA with a construct encoding LHR-ACD as compared to LHR-ABCD [25], and a second showing a 2-fold decrease with an LHR-AC chimera [61]. Removal of either the remaining LHR-B/-C or LHR-A domains further reduces the DAA dramatically (Table 5), with LHR-BC and LHR-A alone showing only residual activity compared to LHR-ABCD, and LHR-AC displaying 10-fold better DAA than LHR-A alone [61].

## 6. Co-Factor Activity of CR1

### 6.1. General and Comparative

Both C3b and C4b can be cleaved by complement Factor I in the presence of CR1, a process referred to as co-factor activity (CFA) and one that was first described for CR1 more than 40 years ago [20,32,59,64]. Subsequent experiments were able to confirm the CFA of CR1 by demonstrating its ability to promote the cleavage of C3b to iC3b and then to C3dg, as well as the cleavage of methylamine-treated C4 (C4-ma; a C4b analog) to C4c and C4d [16,24]. The CFA of CR1 is of particular interest when comparisons between its activity to C3b and C4b are made. Not only does C4b cleavage proceed more slowly than C3b in the presence of equal amounts of ligand, CR1, and Factor I [42,65], but sCR1 has a significantly stronger CFA IC_50_ for C3b (0.8 nM) than it does for C3b (15 nM) [66]. One study showed that Factor I with sCR1 took 1hr to cleave 50% of a C3b preparation compared to 4hr to cleave 50% of a similar amount of C4b [42]. These data demonstrate the relative strength of CR1 CFA for C3b compared to the much weaker CFA for C4b and correlate well with the ligand binding properties of CR1 for C3b and 4b as described above (Table 1 and Table 2).

### 6.2. Domain Contribution

The contribution of CR1 LHR domains to C3b CFA appears reasonably well established but is by no means definitive. The uncertainty is driven primarily by the lack of discrimination in many experiments between the use of monomeric and dimeric ligand, and the somewhat qualitative nature of the assays used (e.g., visualization of cleavage products on protein gels), which could impact data interpretation. However, for C3b CFA, it was shown using both single domains and LHR domain deletion mutants that there is a clear contribution of the LHR-B and LHR-C domains and no contribution of the LHR-D domain [34,43,45,67]. This makes mechanistic sense since CR1 would need to bind ligand to allow Factor I-mediated cleavage to occur. LHR-C point mutants that reduce C3b binding also reduce CFA [67]. It appears that one of the two C3b binding sites can be deleted with minimal loss of C3b CFA, as demonstrated by comparing the relative CFA of an LHR-ACD mutant (8 nM IC_50_) with LHR-ABCD (5 nM IC_50_) [25]. It appears from the results of several studies that the LHR-A domain also contributes weak C3b CFA, although the degree varies from study to study [27,42,45,66]. Given the ambiguity of the binding data of LHR-A to C3b described earlier, we would argue that the observed C3b CFA for LHR-A indirectly suggests that LHR-A could indeed bind C3b and that the weakness of binding is reflected in its weak CFA.

The data pertaining to the role of the CR1 LHR domains in C4b CFA are harder to interpret in light of previously described ligand binding data (Figure 1B) and are somewhat conflicting for LHR-A (containing the main C4b binding site). For those studies that show C4b CFA for LHR-A [36,42,66], data suggest only modest activity with one study showing a threefold decrease in CFA of a construct encoding SCR1–4 of LHR-A compared to sCR1 [66] and another study showing that conversion of the C4b binding site in LHR-A to a C3b binding site by amino acid substitution produced stronger CFA [42]. In the latter study, separate mutations at G76 and Y175 in LHR-A that abrogated C4b binding also abrogated C4b CFA. In contrast to these data, Scesney et al. [27] showed that an sCR1 truncation mutant lacking LHR-A had similar C4b CFA compared to sCR1 alone, and Yazdanbakhsh et al. [45] showed using purified His-tagged LHR-A domains that both LHR-A and LHR-D contained no C4b CFA at all. Studies examining the role of LHR-B and -C in mediating C4b CFA have demonstrated unambiguous involvement with the bulk of activity residing in these domains [27,36,43,45]. Due to the lack of correlation with ligand binding, data pertaining to the relative involvement of LHR-A, -B, and -C in mediating C4b CFA are difficult to understand. For example, why would LHR-B and -C, which both have weak C4b binding (Figure 1B), show stronger C4b CFA than LHR-A, which has a higher affinity to C4b? More work needs to be undertaken to answer these questions so that the ligand binding and DAA data can be aligned with the CFA data into a working mechanistic model.

## 7. Domain Contribution to CR1-Mediated Complement Pathway Inhibition

Until recently, the literature surrounding the role of LHR or SCR domains in mediating complement pathway inhibition as measured by hemolytic activity has been limited. Two studies examined individual and combinations of LHR domains for their ability to inhibit hemolytic activity, but no dose responses were generated since only single, high (1 uM) protein concentrations were used [17,26,45]. However, sufficient data were generated to determine that LHR-A, -B, -C, but not -D, contributed to the overall hemolytic inhibitory activity of CR1. The data also showed that the removal of LHR-A resulted in a decrease in hemolytic inhibitory activity compared to LHR-ABCD, and the removal of both LHR-A and -B further reduced activity. A separate study looking specifically at the inhibition of classical pathway hemolytic activity also showed that deletion of LHR-A from a construct encoding LHR-ABCD reduced activity, although no difference was observed in an alternative pathway hemolytic assay [27].

Other studies examined the requirement of SCR domains to block lytic activity. Makrides et al. [26] showed that SCR1–4 of LHR-A and SCR15–18 of LHR-C could inhibit CHO cell lysis; this activity was abrogated when only SCR1–2 or SCR15–16 were tested. A subsequent experiment by the same group showed that SCR1–4 was approximately 1.3-fold more potent than SCR1–3 and up to 200-fold more potent than SCR1–2 in classical pathway hemolytic assays. However, SCR1–3 was still found to be 300-fold and 20-fold less potent than sCR1 in classical and alternative pathway hemolytic assays, respectively [66]. Spacing between the ligand binding sites in LHR-A and -B is important for inhibitory hemolytic activity as well, with experiments showing that removing SCR4–7 from a construct encoding LHR-AC (i.e., SCR1–3,15–21) effectively rendered the construct as potent as LHR-A alone [62]. Taken together, the studies described above show inhibition of hemolytic activity requires the contribution of LHR-A, -B, and -C domains, and that the same sites used for ligand binding, DAA, and CFA are also required for potency. Unfortunately, these studies also suffer from the lack of quantitative data, and the specific roles of LHR domains in mediating the lectin and alternative pathways were not elucidated in detail.

## 8. CSL040 and Its Mechanism of Action

CSL040 is a truncation variant of sCR1 containing the LHR-A, -B, and -C domains, and with the C-terminal LHR-D domain deleted [19]. The in vitro and in vivo functions of CSL040 have been described elsewhere [18,68,69], but what is of interest for the purposes of this review is that the identification of CSL040 involved the generation and comparative potency testing of deletion variants of sCR1 that allow us to understand the contribution of the individual CR1 LHR domains to in vitro potency in some detail [18] (Table 6).

When recombinant soluble versions of LHR-A, -B, and -C were assessed singly in vitro for their ability to inhibit classical pathway hemolytic activity, all three domains were separately shown to be weakly inhibitory [18]. LHR-A was more than 100-fold less active than CSL040, and LHR-B/-C was more than 1000-fold less active (Table 6). This suggests that the greater affinity of C4b for LHR-A compared to LHR-B/-C (Figure 1B) is reflected in its greater contribution to classical pathway inhibitory activity, but also demonstrates that single LHR domains are not sufficient for full activity and a bivalent interaction with multiple domains is required. For alternative pathway hemolytic activity, LHR-A, -B, and -C each have more than 50-fold lower potency than CSL040 but have similar potency to each other (Table 6). Given the differences in C3b affinity for LHR-A compared to LHR-B/-C (Figure 1A), this observation suggests that the contribution of the LHR-A domain to alternative pathway activity appears to involve additional complexity—this will need to be explored in greater detail. The LHR-D domain has no role in the inhibition of hemolytic activity [18], confirming earlier observations [17,26,45]. However, its removal from sCR1 to generate LHR-ABC/CSL040 resulted in increased in vitro and in vivo potency [18]. The reasons for this have been discussed [19], but it is worth highlighting that any biological effect resulting from the interaction of the LHR-D domain of CR1 with the opsonins MBL, C1q, and/or L- or H- ficolin does not appear to impact the inhibition of hemolytic activity mediated by C3b/C4b, and in fact appears to be somewhat deleterious.

Adding the second, adjacent LHR domain (LHR-A to LHR-B, or LHR-B to LHR-C) to generate LHR-AB and LHR-BC provides a significant and synergistic increase in potency to both classical and alternative pathway hemolytic activity (Table 6) [18]. Interestingly, the LHR-BC combination provided a 50–100-fold increase in classical pathway inhibition, but only up to a 5-fold increase in alternative pathway inhibition compared to single LHR domains (Table 6). Adding the LHR-A domain to LHR-BC (or LHR-C to LHR-AB) to generate CSL040/LHR-ABC provided a further 10-fold improvement to both classical and lectin pathway hemolytic inhibitory activity (Table 6). Although not reproduced here, experiments using Wieslab assays as an alternate method of assessing in vitro potency of the same LHR domain variants yielded similar results, and assay data specific for the complement lectin pathway was comparable to that of the classical pathway [18]. These experiments show that all three LHR domains (-A, -B, and -C) are required from each CR1 molecule for full potency in vitro and that the sequential addition of each LHR domain provides a synergistic rather than additive increase in potency. LHR-AB and LHR-BC are likely to interact bivalently and with high affinity to homo- and hetero-dimers of C3b and/or C4b present in C5 convertases (as well as to monomeric ligand present in C3 convertases with low affinity). In contrast, the single domain LHR-A, -B, or -C molecules would only be able to bind monomeric ligand. How a molecule such as CSL040 specifically interacts with its ligands to generate a degree of inhibition of all three complement pathways an order of magnitude better than CR1 variants LHR-AB or LHR-BC is a question that remains to be answered. Perhaps the third LHR domain in CSL040 can additionally interact with and provide DAA and CFA on a separate C3 convertase.

The final aspect of the molecular mechanism of CSL040 to be discussed is its significantly increased potency for an alternative pathway compared to the classical/lectin pathways. This phenomenon was first observed in vitro [18] but became more apparent following ex vivo pharmacodynamic assays conducted following the administration of CSL040 to either rats or non-human primates [69]. The increased duration of ex vivo alternative pathway inhibition by CSL040 compared to the classical/lectin pathway can be explained mechanistically from the DAA and CFA data described above showing that sCR1 has >10-fold greater affinity to C3b than to C4b [36], can block the binding of C3b to E-CR1 10-fold more strongly than C4b [24], has a 10-fold higher DAA of the alternative pathway convertases compared to those of the classical/lectin pathway [25], and is also much more effective in mediating C3b CFA than C4b CFA [66].

## 9. Conclusions

**Figure 2:**
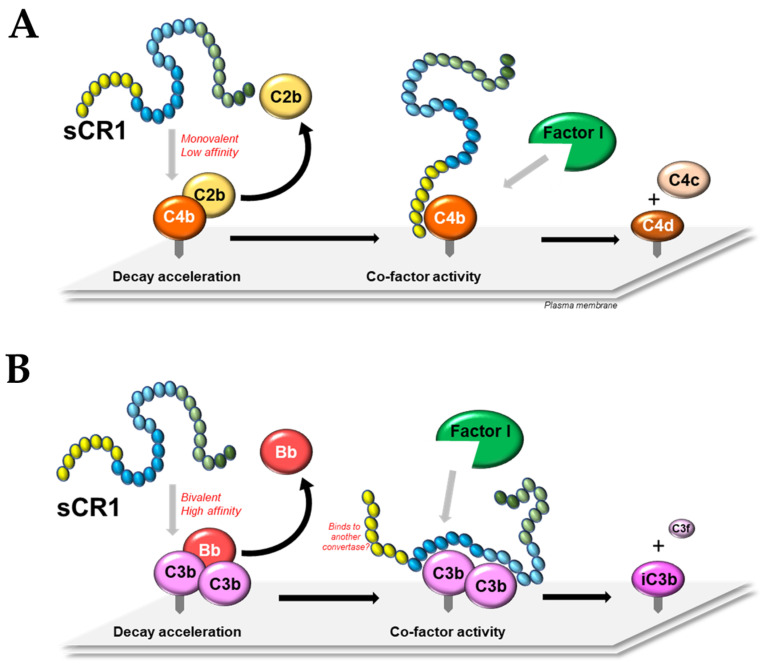
A model of the molecular mechanisms of soluble CR1. (**A**) Soluble CR1 (sCR1) binds to the CP/LP C3 convertase C4bC2b monovalently with low affinity, disrupting the interaction between membrane-associated C4b and C2b (decay acceleration activity). In this panel, LHR-A (yellow) of sCR1 binds C4b, allowing Factor I to cleave C4b into C4c and C4d (co-factor activity). For the AP C3 convertase (not shown), the mechanism is similar except that LHR-B or -C of sCR1 (blue) now interacts with C3b and displaces Bb from the C3bBb convertase. Factor I then cleaves C3b into iC3b and C3f. (**B**) Decay acceleration of the AP C5 convertase C3bBbC3b is mediated by the bivalent and high-affinity interaction of sCR1 with homodimeric C3b, displacing Bb from the convertase. This allows cleavage of C3b into iC3b and C3f by Factor I. For DAA of the CP/LP C5 convertase (not shown), this process is similar but with binding of sCR1 to heterodimeric C4bC3b and displacement of C2b, followed by cleavage of C3b/C4b by Factor I as described above. Following binding of sCR1 to C5 convertases, the unbound C3b or C4b binding site could potentially interact with additional convertases to further increase its potency.

There are still several gaps in our understanding of the molecular mechanisms underlying CR1 function, but there is sufficient information to propose a working model, as shown in Figure 2. In order to inhibit complement activation and downstream lytic activity, CR1 must bind its ligands C3b and C4b. By doing so it competitively displaces C2b or Bb from the nascent convertases by decay acceleration activity. The interaction with monomeric or dimeric ligand will determine whether the DAA is against a C3 convertase (C4bC2b or C3bBb) or a C5 convertase (C4bC2bC3b or C3bBbC3b), respectively. CR1 binding to monomeric ligand appears to be monovalent and low(er) affinity, but bivalent and high(er) affinity to dimeric ligand, with different domains within a single CR1 molecule contributing to binding depending on the type of dimer: C3b-C3b, C3b-C4b or C4b-C4b. Spacing between ligand binding sites on CR1 is important—this allows the appropriate degree of SCR domain folding for contact to occur. The presence of a third C3b/C4b binding site in CR1 adds further complexity to this interaction, which remains to be elucidated. The binding of CR1 to C3b/C4b also provides an opportunity for CFA, with Factor I processing these CR1-bound activated complement fragments into their inactive forms by proteolytic cleavage. The relative strength of the interaction of CR1 with C3b compared to C4b determines the relative potency of this molecule to the classical, lectin, and alternative pathways, with inhibition of the latter being the most effective.

## Figures and Tables

**Table 1:** Binding of CR1 to C3b.

Affinity to C3b Dimer (nM)	Affinity to C3b Monomer (nM)	Method Used	CR1 Source	References
9–16	No binding	Radioligand binding assay	E-CR1	Arnaout et al., 1981 [22]
4.62	No binding ^a^	Radioligand binding assay	E-CR1	Arnaout et al., 1983 [23]
1.3 ^b^	>100-fold weaker ^c^	Radioligand binding/competition	sCR1 and E-CR1	Wiesman et al., 1990 [24]
10 ^b^	1000 ^b^	Radioligand binding/competition	sCR1 and E-CR1	Wong and Farrell 1991 [25]
1.0	Not measured	Radioligand binding assay	Expressed on K562 cells	Kalli et al., 1991 [16]
19	200	Radioligand binding assay	E-CR1	Makrides et al., 1992 [26]
12–19	800	Radioligand binding assay	Expressed on CHO cells	Makrides et al., 1992 [26]
30 ^b^	600 ^b^	Radioligand binding/competition	sCR1 and E-CR1	Scesney et al., 1996 [27]
17.9–40.6	Not measured	SPR	sCR1	Klickstein et al., 1997 [28]
140 ^d^	Not measured	SPR	sCR1	Alcorlo et al., 2011 [29]
69 ^e^		SPR	sCR1	Schramm et al., 2015 [30]
385.7 ^e^		SPR	sCR1	Wymann et al., 2021 [18]

^a^ Affinity of 35.6 nM measured at low (non-physiological) ionic strength; ^b^ estimates of affinity based on concentration of sCR1 needed for 50% inhibition of ^125^I-C3b binding to E-CR1. ^c^ Compared to dimer. ^d^ Steady-state estimate of affinity. ^e^ Affinity measured against plasma-derived C3b containing a mixture of monomeric and dimeric C3b; displayed values are therefore not aligned under either column. E-CR1: erythrocyte-expressed CR1. CHO: Chinese hamster ovary. SPR: surface plasmon resonance. sCR1: soluble CR1.

**Table 3:** The contribution of CR1 domains to classical pathway C3 Convertase (C4bC2b) DAA.

LHR-A	LHR-B	LHR-C	LHR-D	CP C3 DAA Activity Relative to LHR-ABCD
				60%, 43% ^a^
				12%
				8%, 10% ^b^
				0%
				100%
				100%
				8%
Required	Either site required		Not Required	

The filled cells along each row correspond to constructs encoding the relevant LHR domains used experimentally to determine the LHR contribution to DAA, and the right-hand column shows the DAA(s) of that construct relative to LHR-ABCD, based on the available literature [25,61,62]. ^a^ Percentage DAA relative to LHR-AC. ^b^ Percentage DAA relative to LHR-A. The bottom row shows the overall LHR domain requirement for DAA that can be inferred from the experimental data. Lectin pathway DAA is assumed to be identical to that of the classical pathway (CP), given the convertases used for both pathways are the same.

**Table 4:** The contribution of CR1 domains to classical pathway C5 Convertase (C4bC2bC3b) DAA.

LHR-A	LHR-B	LHR-C	LHR-D	CP C5 DAA Activity Relative to LHR-ABCD
				0.5%, 1% ^a^
				3%
				95% ^b^
				100%
Required	Either site required		Not Required	

The filled cells along each row correspond to constructs encoding the relevant LHR domains used experimentally to determine the LHR contribution to DAA, and the right-hand column shows the DAA(s) of that construct relative to LHR-ABCD, based on the available literature [25,61,62]. ^a^ Percentage DAA relative to LHR-AC. ^b^ A separate experiment showed 100-fold greater activity relative to LHR-A. The bottom row shows the overall LHR domain requirement for DAA that can be inferred from the experimental data. Lectin pathway DAA is assumed to be identical to that of the classical pathway.

**Table 5:** The contribution of CR1 domains to alternative pathway C5 Convertase (C3bBbC3b) DAA.

LHR-A	LHR-B	LHR-C	LHR-D	AP C5 DAA Activity Relative to LHR-ABCD
				0.5%, 10% ^a^
				0%
				0%
				0%
				50%
				30-fold weaker
				0.6%
Required	Required	Required	Not Required	

The filled cells along each row correspond to constructs encoding the relevant LHR domains used experimentally to determine the LHR contribution to alternative pathway (AP) C5 DAA, and the right-hand column shows the DAA(s) of that construct relative to LHR-ABCD, based on the available literature [25,61,62]. ^a^ Percentage DAA relative to LHR-AC. The bottom row shows the overall LHR domain requirement for DAA that can be inferred from the experimental data.

**Table 6:** Comparative hemolytic data for LHR deletion variants of sCR1.

CR1 Domain(s)	Number Of C3b/C4b Binding Sites	Classical IC_50_ (nM) ± S.D.	Alternative IC_50_ (nM) ± S.D.
LHR-ABC/CSL040	3	0.42 ± 0.10	0.90 ± 0.54
LHR-AB	2	4.61 ± 1.85	13.15 ± 2.18
LHR-BC	2	10.08 ± 5.52	12.48 ± 4.30
LHR-A	1	68.38 ± 9.52	49.87 ± 24.76
LHR-B	1	959.37 ± 243.92	63.38 ± 31.03
LHR-C	1	733.10 ± 203.47	66.36 ± 18.10
LHR-D	0	No Activity	No Activity

Selected IC_50_ values reproduced from Wymann et al., 2021 [18], from complement classical and alternative pathway hemolytic assays where the indicated variants of sCR1 containing 1, 2, or 3 LHR domains were analyzed for comparative potency. Values shown are the mean ± standard deviation (S.D.).

## Data Availability

No data were used for the research described in the article.

## Abbreviations

CR1, complement receptor 1; DAA, decay acceleration activity; CFA, co-factor activity; E-CR1, erythrocyte CR1; SCR, short consensus repeat; LHR, long homologous repeat; SPR, surface plasmon resonance; MBL, mannose-binding lectin; CHO, Chinese hamster ovary.

## References

[1] Fearon D.T. (1980). Identification of the membrane glycoprotein that is the C3b receptor of the human erythrocyte, polymorphonuclear leukocyte, B lymphocyte, and monocyte. J. Exp. Med..

[2] Ross G.D., Lambris J.D. (1982). Identification of a C3bi-specific membrane complement receptor that is expressed on lymphocytes, monocytes, neutrophils, and erythrocytes. J. Exp. Med..

[3] Ahearn J.M., Fearon D.T. (1989). Structure and function of the complement receptors, CR1 (CD35) and CR2 (CD21). Adv. Immunol..

[4] Lublin D.M., Griffith R.C., Atkinson J.P. (1986). Influence of glycosylation on allelic and cell-specific Mr variation, receptor processing, and ligand binding of the human complement C3b/C4b receptor. J. Biol. Chem..

[5] Lachmann P.J. (1990). Biological functions of the complement system. Biochem. Soc. Trans..

[6] Ricklin D., Hajishengallis G., Yang K., Lambris J.D. (2010). Complement: A key system for immune surveillance and homeostasis. Nat. Immunol..

[7] Kolev M., Le Friec G., Kemper C. (2014). Complement—Tapping into new sites and effector systems. Nat. Rev. Immunol..

[8] Lachmann P.J. (2009). The amplification loop of the complement pathways. Adv. Immunol..

[9] Pangburn M.K. (2022). Initiation of the alternative pathway of complement and the history of “tickover”. Immunol. Rev..

[10] Wagner E., Frank M.M. (2010). Therapeutic potential of complement modulation. Nat. Rev. Drug Discov..

[11] Krych-Goldberg M., Atkinson J.P. (2001). Structure-function relationships of complement receptor type 1. Immunol. Rev..

[12] Furtado P.B., Huang C.Y., Ihyembe D., Hammond R.A., Marsh H.C., Perkins S.J. (2008). The partly folded back solution structure arrangement of the 30 SCR domains in human complement receptor type 1 (CR1) permits access to its C3b and C4b ligands. J. Mol. Biol..

[13] Weis J.H., Morton C.C., Bruns G.A., Weis J.J., Klickstein L.B., Wong W.W., Fearon D.T. (1987). A complement receptor locus: Genes encoding C3b/C4b receptor and C3d/Epstein-Barr virus receptor map to 1q32. J. Immunol..

[14] Klickstein L.B., Wong W.W., Smith J.A., Weis J.H., Wilson J.G., Fearon D.T. (1987). Human C3b/C4b receptor (CR1). Demonstration of long homologous repeating domains that are composed of the short consensus repeats characteristics of C3/C4 binding proteins. J. Exp. Med..

[15] Liu D., Niu Z.X. (2009). The structure, genetic polymorphisms, expression and biological functions of complement receptor type 1 (CR1/CD35). Immunopharmacol. Immunotoxicol..

[16] Kalli K.R., Hsu P.H., Bartow T.J., Ahearn J.M., Matsumoto A.K., Klickstein L.B., Fearon D.T. (1991). Mapping of the C3b-binding site of CR1 and construction of a (CR1)2-F(ab’)2 chimeric complement inhibitor. J. Exp. Med..

[17] Mqadmi A., Abdullah Y., Yazdanbakhsh K. (2005). Characterization of complement receptor 1 domains for prevention of complement-mediated red cell destruction. Transfusion.

[18] Wymann S., Dai Y., Nair A.G., Cao H., Powers G.A., Schnell A., Martin-Roussety G., Leong D., Simmonds J., Lieu K.G. (2021). A novel soluble complement receptor 1 fragment with enhanced therapeutic potential. J. Biol. Chem..

[19] Hardy M.P., Rowe T., Wymann S. (2022). Soluble Complement Receptor 1 Therapeutics. J. Immunol. Sci..

[20] Fearon D.T. (1979). Regulation of the amplification C3 convertase of human complement by an inhibitory protein isolated from human erythrocyte membrane. Proc. Natl. Acad. Sci. USA.

[21] Karthikeyan G., Baalasubramanian S., Seth S., Das N. (2007). Low levels of plasma soluble complement receptor type 1 in patients receiving thrombolytic therapy for acute myocardial infarction. J. Thromb. Thrombolysis.

[22] Arnaout M.A., Melamed J., Tack B.F., Colten H.R. (1981). Characterization of the human complement (c3b) receptor with a fluid phase C3b dimer. J. Immunol..

[23] Arnaout M.A., Dana N., Melamed J., Medicus R., Colten H.R. (1983). Low ionic strength or chemical cross-linking of monomeric C3b increases its binding affinity to the human complement C3b receptor. Immunology.

[24] Weisman H.F., Bartow T., Leppo M.K., Marsh H.C., Carson G.R., Concino M.F., Boyle M.P., Roux K.H., Weisfeldt M.L., Fearon D.T. (1990). Soluble human complement receptor type 1: In vivo inhibitor of complement suppressing post-ischemic myocardial inflammation and necrosis. Science.

[25] Wong W.W., Farrell S.A. (1991). Proposed structure of the F’ allotype of human CR1. Loss of a C3b binding site may be associated with altered function. J. Immunol..

[26] Makrides S.C., Scesney S.M., Ford P.J., Evans K.S., Carson G.R., Marsh H.C. (1992). Cell surface expression of the C3b/C4b receptor (CR1) protects Chinese hamster ovary cells from lysis by human complement. J. Biol. Chem..

[27] Scesney S.M., Makrides S.C., Gosselin M.L., Ford P.J., Andrews B.M., Hayman E.G., Marsh H.C. (1996). A soluble deletion mutant of the human complement receptor type 1, which lacks the C4b binding site, is a selective inhibitor of the alternative complement pathway. Eur. J. Immunol..

[28] Klickstein L.B., Barbashov S.F., Liu T., Jack R.M., Nicholson-Weller A. (1997). Complement receptor type 1 (CR1, CD35) is a receptor for C1q. Immunity.

[29] Alcorlo M., Martinez-Barricarte R., Fernandez F.J., Rodriguez-Gallego C., Round A., Vega M.C., Harris C.L., de Cordoba S.R., Llorca O. (2011). Unique structure of iC3b resolved at a resolution of 24 A by 3D-electron microscopy. Proc. Natl. Acad. Sci. USA.

[30] Schramm E.C., Roumenina L.T., Rybkine T., Chauvet S., Vieira-Martins P., Hue C., Maga T., Valoti E., Wilson V., Jokiranta S. (2015). Mapping interactions between complement C3 and regulators using mutations in atypical hemolytic uremic syndrome. Blood.

[31] Dobson N.J., Lambris J.D., Ross G.D. (1981). Characteristics of isolated erythrocyte complement receptor type one (CR1, C4b-C3b receptor) and CR1-specific antibodies. J. Immunol..

[32] Ross G.D., Lambris J.D., Cain J.A., Newman S.L. (1982). Generation of three different fragments of bound C3 with purified factor I or serum. I. Requirements for factor H vs. CR1 cofactor activity. J. Immunol..

[33] Ross G.D. (1980). Analysis of the different types of leukocyte membrane complement receptors and their interaction with the complement system. J. Immunol. Methods.

[34] Klickstein L.B., Bartow T.J., Miletic V., Rabson L.D., Smith J.A., Fearon D.T. (1988). Identification of distinct C3b and C4b recognition sites in the human C3b/C4b receptor (CR1, CD35) by deletion mutagenesis. J. Exp. Med..

[35] Pangburn M.K. (1986). Differences between the binding sites of the complement regulatory proteins DAF, CR1, and factor H on C3 convertases. J. Immunol..

[36] Reilly B.D., Makrides S.C., Ford P.J., Marsh H.C., Mold C. (1994). Quantitative analysis of C4b dimer binding to distinct sites on the C3b/C4b receptor (CR1). J. Biol. Chem..

[37] Belt K.T., Carroll M.C., Porter R.R. (1984). The structural basis of the multiple forms of human complement component C4. Cell.

[38] Clemenza L., Isenman D.E. (2004). The C4A and C4B isotypic forms of human complement fragment C4b have the same intrinsic affinity for complement receptor 1 (CR1/CD35). J. Immunol..

[39] Gatenby P.A., Barbosa J.E., Lachmann P.J. (1990). Differences between C4A and C4B in the handling of immune complexes: The enhancement of CR1 binding is more important than the inhibition of immunoprecipitation. Clin. Exp. Immunol..

[40] Reilly B.D., Mold C. (1997). Quantitative analysis of C4Ab and C4Bb binding to the C3b/C4b receptor (CR1, CD35). Clin. Exp. Immunol..

[41] Prohaska R., Adolf G.R. (1987). Characterization of the human erythrocyte complement receptor CR1 (C3b receptor) by epitope mapping. Immunobiology.

[42] Krych M., Clemenza L., Howdeshell D., Hauhart R., Hourcade D., Atkinson J.P. (1994). Analysis of the functional domains of complement receptor type 1 (C3b/C4b receptor; CD35) by substitution mutagenesis. J. Biol. Chem..

[43] Krych M., Hauhart R., Atkinson J.P. (1998). Structure-function analysis of the active sites of complement receptor type 1. J. Biol. Chem..

[44] Krych M., Hourcade D., Atkinson J.P. (1991). Sites within the complement C3b/C4b receptor important for the specificity of ligand binding. Proc. Natl. Acad. Sci. USA.

[45] Yazdanbakhsh K., Kang S., Tamasauskas D., Sung D., Scaradavou A. (2003). Complement receptor 1 inhibitors for prevention of immune-mediated red cell destruction: Potential use in transfusion therapy. Blood.

[46] Smith B.O., Mallin R.L., Krych-Goldberg M., Wang X., Hauhart R.E., Bromek K., Uhrin D., Atkinson J.P., Barlow P.N. (2002). Structure of the C3b binding site of CR1 (CD35), the immune adherence receptor. Cell.

[47] Korb L.C., Ahearn J.M. (1997). C1q binds directly and specifically to surface blebs of apoptotic human keratinocytes: Complement deficiency and systemic lupus erythematosus revisited. J. Immunol..

[48] Sontheimer R.D., Racila E., Racila D.M. (2005). C1q: Its functions within the innate and adaptive immune responses and its role in lupus autoimmunity. J. Investig. Dermatol..

[49] Tas S.W., Klickstein L.B., Barbashov S.F., Nicholson-Weller A. (1999). C1q and C4b bind simultaneously to CR1 and additively support erythrocyte adhesion. J. Immunol..

[50] Jacquet M., Cioci G., Fouet G., Bally I., Thielens N.M., Gaboriaud C., Rossi V. (2018). C1q and Mannose-Binding Lectin Interact with CR1 in the Same Region on CCP24-25 Modules. Front. Immunol..

[51] Ghiran I., Barbashov S.F., Klickstein L.B., Tas S.W., Jensenius J.C., Nicholson-Weller A. (2000). Complement receptor 1/CD35 is a receptor for mannan-binding lectin. J. Exp. Med..

[52] Jacquet M., Lacroix M., Ancelet S., Gout E., Gaboriaud C., Thielens N.M., Rossi V. (2013). Deciphering complement receptor type 1 interactions with recognition proteins of the lectin complement pathway. J. Immunol..

[53] Kilpatrick D.C., Chalmers J.D. (2012). Human L-ficolin (ficolin-2) and its clinical significance. J. Biomed. Biotechnol..

[54] Emlen W., Burdick G., Carl V., Lachmann P.J. (1989). Binding of model immune complexes to erythrocyte CR1 facilitates immune complex uptake by U937 cells. J. Immunol..

[55] Nardin A., Lindorfer M.A., Taylor R.P. (1999). How are immune complexes bound to the primate erythrocyte complement receptor transferred to acceptor phagocytic cells?. Mol. Immunol..

[56] Janssen B.J., Christodoulidou A., McCarthy A., Lambris J.D., Gros P. (2006). Structure of C3b reveals conformational changes that underlie complement activity. Nature.

[57] Park H.J., Guariento M., Maciejewski M., Hauhart R., Tham W.H., Cowman A.F., Schmidt C.Q., Mertens H.D., Liszewski M.K., Hourcade D.E. (2014). Using mutagenesis and structural biology to map the binding site for the Plasmodium falciparum merozoite protein PfRh4 on the human immune adherence receptor. J. Biol. Chem..

[58] Forneris F., Wu J., Xue X., Ricklin D., Lin Z., Sfyroera G., Tzekou A., Volokhina E., Granneman J.C., Hauhart R. (2016). Regulators of complement activity mediate inhibitory mechanisms through a common C3b-binding mode. EMBO J..

[59] Iida K., Nussenzweig V. (1981). Complement receptor is an inhibitor of the complement cascade. J. Exp. Med..

[60] Takata Y., Kinoshita T., Kozono H., Takeda J., Tanaka E., Hong K., Inoue K. (1987). Covalent association of C3b with C4b within C5 convertase of the classical complement pathway. J. Exp. Med..

[61] Krych-Goldberg M., Hauhart R.E., Subramanian V.B., Yurcisin B.M., Crimmins D.L., Hourcade D.E., Atkinson J.P. (1999). Decay accelerating activity of complement receptor type 1 (CD35). Two active sites are required for dissociating C5 convertases. J. Biol. Chem..

[62] Krych-Goldberg M., Hauhart R.E., Porzukowiak T., Atkinson J.P. (2005). Synergy between two active sites of human complement receptor type 1 (CD35) in complement regulation: Implications for the structure of the classical pathway C3 convertase and generation of more potent inhibitors. J. Immunol..

[63] Kinoshita T., Takata Y., Kozono H., Takeda J., Hong K.S., Inoue K. (1988). C5 convertase of the alternative complement pathway: Covalent linkage between two C3b molecules within the trimolecular complex enzyme. J. Immunol..

[64] Medof M.E., Iida K., Mold C., Nussenzweig V. (1982). Unique role of the complement receptor CR1 in the degradation of C3b associated with immune complexes. J. Exp. Med..

[65] Medof M.E., Nussenzweig V. (1984). Control of the function of substrate-bound C4b-C3b by the complement receptor Cr1. J. Exp. Med..

[66] Mossakowska D., Dodd I., Pindar W., Smith R.A. (1999). Structure-activity relationships within the N-terminal short consensus repeats (SCR) of human CR1 (C3b/C4b receptor, CD35): SCR 3 plays a critical role in inhibition of the classical and alternative pathways of complement activation. Eur. J. Immunol..

[67] Smith R.A. (2002). Targeting anticomplement agents. Biochem. Soc. Trans..

[68] Bongoni A.K., Vikstrom I.B., McRae J.L., Salvaris E.J., Fisicaro N., Pearse M.J., Wymann S., Rowe T., Morelli A.B., Hardy M.P. (2021). A potent truncated form of human soluble CR1 is protective in a mouse model of renal ischemia-reperfusion injury. Sci. Rep..

[69] Wymann S., Mischnik M., Leong D., Ghosh S., Tan X., Cao H., Kuehnemuth B., Powers G.A., Halder P., de Souza M.J. (2022). Sialylation-dependent pharmacokinetics and differential complement pathway inhibition are hallmarks of CR1 activity in vivo. Biochem. J..

